# Sound Practice, Sound Philosophy

**Published:** 1873-03

**Authors:** M. S. Dean


### ARTICLE II.

### *Sound Practice, Sound Philosophy.*

BY M. S. DEAN, D. D. S.

Our practice, as professional men, to be successful in its results, must be based upon sound philosophy. By this, I mean the knowledge of those principles and laws that govern the materials or agents *with* which we operate, and also those *upon* which we operate.

Unless we understand these laws, our practice is simply imitative, or guess work—unless we are familiar with these principles, we shall continually encounter difficulties, that a better knowledge of them would lead us to easily avoid. Anatomy, Physiology and Pathology are of little avail in the practice of our profession, if we lack the essential principles of this branch of physical science. We must understand the properties and laws of the dead matter with which we deal, as well as those of the living. The principles of philosophy run through the whole gamut of our practice, even from the material and force of the mallet up to the most obscure and difficult disease of the oral cavity. I am amazed when I come to consider the magnitude of this subject, and mortified when I think how insignificant my efforts must appear in comparison with the title—yet, this title affords me one great advantage, and that is, an unlimited range over the whole theory and practice of our profession. I know I ought to apologize to you for not coming sooner to the practical questions involved in this subject, and I fear that the course I have pursued may have already reminded you of Hermanus Van Clottercop, the builder of the large stone church at Rotterdam, as related by Irving, in *Knickerbocker's New York.*

The first object that meets my eye as I enter the borders of this great field is the mallet; a very simple instrument, apparently, but I will stop a moment to examine its characteristics.

I remember to have read somewhere a discussion in regard to the splitting of the bicuspid tooth in filling a cavity in its grinding surface. From the tone of the remarks the reader must believe that the prevalent idea was, in this Society, that the heavier the mallet the more liable it would be to produce this fracture. Now, one familiar with the laws of impact would not need this lesson to teach him the fact that the quick stroke of a light mallet would be much more liable to split this tooth than the heavier one, both having the same force of impact. We all recognize the fact that with a light steel or wooden mallet we can shiver to atoms a small glass ball that is tossed in the air. Whereas, with a six ounce lead mallet we should propel the ball uninjured quite a distance. The heavier mallet may have the greater momentum, yet the one which shivers the ball will have the greatest *vis-viva.*

Again. If we have an extracted bicuspid which we wish to split with a chisel, we should take our lighter rather than our heavy mallet to give the stroke. With this we should split the tooth without driving the roots into a pine table upon which it rests; but if we use the heavier it will imbed the roots a considerable distance before the cleavage can be accomplished. This shows us that greater force can be given with a heavy mallet before splitting the bicuspid, than with the lighter one. This point I think needs no further elaboration.

I once saw a case where quite a portion of the molar had been split off during the process of filling. The operator was sure it could not have from the blow of the mallet, as he had used a very light wooden one. The philosophy of this operator was not sound, and the fracture of the tooth was the legitimate consequence.

Now the fracture of a tooth is by no means a common result of a light quick blow of the mallet, but it is much more capable of producing this result than the heavy one. So when there is danger to be apprehended, as from the excessive frangibility of the tooth, it is both prudent and philosophical to make use of the heavier. The same evil may result from the use of cuneiform pluggers—*i. e.*, by using those that taper nearly to a point, so as to act as a wedge; or, which would amount to the same thing, by driving the gold too forcibly into a cavity which diminishes from its orifice. Either the gold or the instrument, acting as a wedge, might check the enamel, if it did not split the tooth. This evil might occur from the use of either the light or the heavy mallet, or from hand pressure thus applied, yet the light mallet would be the most liable to produce this unpleasant result. I am led to these remarks from seeing so many bicuspid teeth, with perfect fillings, extending from the posterior to the anterior pit, in which may be discovered a check in the enamel running from the filling to the neck of the tooth. I am aware that these checks are often the result of other causes, but I am also confident that the light mallet may be, and sometimes is, also the cause.

It discovers unsound philosophy in an operator when we find the gold drawn beyond the margin of cavities in the masticating surface of the teeth. We have heard operators speak admiringly of the feat of drawing the gold with the mallet and burnisher *over* the edges of the cavity. I do not believe this practice is in accordance with sound philosophy. Now the thin overlapping metal cannot be beaten over and beyond the margins, so that its entire circumference will be perfectly adapted to the tooth; for the gold has become more elastic from the repeated blows it has received, in order to accomplish this feat, rendering it impossible to bring the gold and tooth into absolute contact. The work may be sufficiently accurate to deceive the eye, yet these spaces, though they be imperceptible, must exist between the overlapping metal and the enamel. These overlappings, however, may not, in most cases, be very *serious* faults, for they will soon be torn away by attrition, yet the margins will not *then* be left as smooth and well defined as though the metal had been trimmed away before the operator dismissed his work.

In filling a *large* crown cavity, with surrounding walls unbroken, it is not sound philosophy to keep the central portion of the filling lower than the peripheral, especially when the work approaches completion. If, during the process of filling, the plug maintains a concave surface, the force applied has a tendency to draw the gold away from the surrounding walls.

It is difficult, if not impossible, in such cases, to beat the metal down against the margins so as to adapt it perfectly in all its parts; for, as before stated, the gold being somewhat elastic, cannot be brought into *absolute* contact throughout the entire periphery of the filling. These can be made perfect only by the principle of wedging. If, after the centre of a *large* filling is nearly or quite complete, there be a small shallow groove left around the margin of the cavity, we can, with a properly adjusted instrument, *wedge* the foil into the groove in such a manner as to make the union absolutely perfect. If in this groove light unannealed foil be used in contact with the margins of the tooth, and perfectly secured by overlapping cohesion, I think it would accord with sound philosophy.

Gold should not be forced *unwillingly*—so to speak—into any portion of a filling where cohesion is important, or against the walls of a cavity. The pellet, or piece of gold, should show its affinity or affection, as Atkinson might say, for the filling upon which it has been placed; for if they do not cohere or hold together until it has received two or three blows, we cannot rely upon permanent cohesion. It *may* scale off afterwards, though malleted down ever so smoothly. The pellet and plug must unite by the "first intention." It must be "love at first sight," and the union must be encouraged and consummated before the affinity is weakened or destroyed. By rolling it from place to place, and partially condensing it, less of its surface can be brought into actual contact with the filling; and the minute projecting filaments, raised by annealing, are smoothed down, rendering it less adhesive. If the philosophy in this case is not clearly presented, the facts will not be disputed.

The area or surface of a plugger point, used for packing, should correspond to a certain degree with the pellet upon which it is used. If the pellet be large and the plugger small, the foil will not be carried before the instrument. It would do little else than punch holes, and make the mass rugged and unyielding. I would not be understood that the plugger-point must necessarily be large enough to cover the entire surface of a large pellet, and yet this would be better practice than to use very sharp-pointed ones. By *introducing* the large pellet with a large plugger, we can bring it smoothly to its place. *Afterwards* we may *condense* with smaller ones to advantage. The deep pits which are such a bugbear in the eyes of the advocates of smooth points, are thus entirely or sufficiently prevented. These "pits" are owing more to the relative small points used in *introducing* the gold, and to the carelessness in condensing, than to the serrations of the instruments used.

I hope the Society will keep in mind the fact I am now referring to the use of *large pellets* in *large cavities;* and also that I neither advocate nor condemn the use of large pellets in such cases, but merely contend that when large masses of foil are used, that they should be packed—not condensed—with large points which will neither perforate nor draw the gold towards the point upon which the instrument is placed. The same reasoning applies, but to a less extent, perhaps, to the use of small sharp points, whether smooth or serrated, in packing the heavier foils, as they tend to draw the foil into a knotty mass, requiring much more mallet force to bring them into a perfectly solid filling. There are, of course, portions of large fillings which are not essential to be made perfectly solid.

In condensing the finishing layers of gold, the light mallet is the most efficient, especially if the last pellets be small, or if we use heavy gold—*provided* we wish to condense merely the surface of the filling—with it we can make the surface dense without affecting materially the mass of metal in the body of the filling. Yet, unless the tooth be a loose one, I should doubt the propriety of using the light mallet on the finishing surface, in the majority of cases, as it is generally more unpleasant to the patient—and the heavier one can be made to accomplish the same results—though in doing it it affects the mass of gold more deeply.

When we can obtain only small retaining points to build a large filling upon, until it can be brought to another point where it can be more securely anchored, the light mallet will be more liable to break the small retainder, or to tear it loose, than the heavier, especially if the force is not directed in a line with the retaining point.

Now, is it sound philosophy to use what are called smooth points in filling teeth? If not, it will be unsound practice. To answer this question we must first consider the advantages claimed by the advocates of this style of instrument. The first and main advantage claimed is that the fillings inserted by them are more dense, that the *surfaces* of the filling will not *before*, nor after use, exhibit a pitted appearance. This is certainly a desirable result to accomplish, but it is by no means an essential one in attaining the object sought in filling the teeth—their preservation. It adds only to the beauty of finish, without increasing its capacity to preserve the teeth from further decay. Now, the chief object in filling a tooth is to restore as permanently as possible, its lost parts, in such a manner as that the juncture of the material used, and the tooth substance be complete and *inaccessible.* It does not lessen the *prospect* of saving the tooth, if the mass of gold in the filling is not perfectly impacted or dense—*provided* the walls of the cavity are absolutely protected, and the filling sufficiently consolidated to resist, without yielding, the repeatedly applied force of mastication. In fact it is more philosophical to leave the central portion *not perfectly* compacted in the very large cavities. Its conductive properties would be lessened, and, what is somewhat important, its expansive and contractible quality would be diminished. So that, if what is claimed for smooth points be true—which I do not admit—that they pack the gold more *densely* in the greater portion of the filling, this compactness does not increase the conservative character of a *very large* filling. Now do not misunderstand me, although I believe that, *in some respects*, a porous filling is better than a perfectly dense one, yet I do not strive to make my fillings porous. On the contrary, I make them as dense as practicable, and still they *are* porous to some extent, and so are yours, whether you use the serrated or non-serrated points. But this is not saying that they are not sufficiently dense. Our fillings are not failing now-a-days from the lack of density, as much as from want of care and judgment in preparing the cavities for their reception ; nor from the lack of density as from the want of contiguity to the walls, especially the marginal walls of the teeth. These should be in absolute contact, if such a state be possible. Density is not a quality to be ignored in a filling, but should by no means be the *chief* aim of the operator. The more essential principles should not be overshadowed nor made secondary to this quality of density.

I may have dwelt upon this point perhaps more than is necessary, but I have seen of late so many dense fillings standing upright in the crown cavities—cohesive solid masses of gold, but distant and respectful to the surrounding walls—that I think I am justified in so doing. This class of failures is now the most prevalent one, and shows conclusively to my mind, that the energies of the operators of to-day are absorbed too exclusively in their efforts to produce this quality in their fillings.

Some of the advocates of the smooth pluggers take grounds that are not only unphilosophical, but ridiculous in the extreme. For instance, we are told that the face of the instrument should be flat and smooth, with sharp edges, and that it is impossible to make the sides of a filling perfect with any other than smooth points. Now is it possible that we can pack the gold more acurately against the concave walls of a tooth with an instrument having a smooth flat surface, than with a serrated convex one? The only place where the smooth points can, perhaps, be made to secure better results than the serrated is upon the surface, or as we near the surface of the filling; and I very much doubt their advantage in this portion of the filling. I believe that a filling can be made practically as dense with the serrated as with the smooth pointed, if the same pains be taken with the former as the latter. When I speak of serrated points, I do not mean points where the serrations are like the tines of a fork—but like Varney's for instance. Now if the fillings introduced with smooth points, after hard usage, do not present a more pitted surface than the unfinished fillings introduced with Varney's pluggers, where equal pains had been taken with both, I am greatly mistaken. I admit that if the surface of the filling could be kept flat, no points could be better adapted to the purpose of making dense fillings than the smooth ones. But this can rarely be done, and, when it is not done, no points are better adapted to shingle, or bridge-over the interstices, and more effectually deceive the operator. And, as before stated, this bridging surface is oftener the cause of a roughened surface in a worn filling than the serrations in the instrument used. Time is certainly lost by using them, and perfection is not certainly gained, and I think it better practice and better philosophy to use serrated points adapted to the portion of the filling introduced, and the form of gold used—using of course shallower serrations as the work approaches completion.

It is asserted by a writer, who advocates the use of the steel mallet and smooth points, that in the operation with these, there is no pain to the patient, no risk of fracturing the enamel—that the fillings are harder and better adapted to the margins of the cavity. The millenium for both the operator and patient has finally dawned if this be true. Whether this magic power resides mostly in the steel mallet or the smooth points, or whether they are equally efficacious in producing these desirable results and in averting the contrary ones the writer has neglected to inform us. It is difficult to understand why the smooth faced instruments should not fracture the enamel as readily as the same instrument serrated like Varney's.

Most dentists place a layer or cushion, as it were, of gold between the instrument and the enamel, before the stroke of the hammer is given, otherwise I see no need of the blow, unless it be for the purpose of fracturing it. If we spread a hair mattress upon a sheet of thin ice, it makes little difference whether we walk over it with a pair of slippers or stogas with nails in the heels, as to the danger of breaking it. So if we pack a pellet of gold against the thin walls of a tooth with a sharp point, the danger of fracture is as great as if the same points had been properly serrated. This being the case, I conclude that the peculiar virtues alluded to by this writer, lie principally in the mallet. For the reasons before given in discussing the relative merits of mallets, I had supposed the *ordinary* steel mallet at least quite as likely to fracture the enamel as the leaden one. If I was correct in this inference—and if this charm does not belong to the smooth pointed pluggers—I must conclude that this merit lies in the *peculiar properties* of the steel of which this wondrous mallet is made. And I am warranted in this conclusion by the assurances of the writer that it is non-vibrative.

Now, seriously, it seems to me that these statements in regard to the non-vibrate mallet and smooth points are, to use a mild term, unphilosophical, yet they are undoubtedly honestly expressed by one whose enthusiasm has got the better of his judgment.

Now, although I find none of my patients who prefer the steel to the leaden mallet, I do not doubt that in certain cases it is more effective. Its *vis viva* is greater in proportion to its momentum, and consequently, in cases where the under jaw shrinks from the blow, and also when the teeth yield in the socket, the lighter steel mallet is somewhat preferable. But it the hand of my assistant, my patients invariably prefer the leaden one.

To Be Continued.

---

